# Modulation of Human Immune Response by Fungal Biocontrol Agents

**DOI:** 10.3389/fmicb.2017.00039

**Published:** 2017-02-03

**Authors:** Cibele Konstantinovas, Tiago A. de Oliveira Mendes, Marcos A. Vannier-Santos, Jane Lima-Santos

**Affiliations:** ^1^Departamento de Ciências Biológicas, Universidade Estadual de Santa CruzIlhéus, Brazil; ^2^Departamento de Bioquímica e Biologia Molecular, Universidade Federal de ViçosaViçosa, Brazil; ^3^Biologia Celular Parasitária, Instituto Gonçalo Moniz, Fundação Oswaldo CruzSalvador, Brazil

**Keywords:** spores, biofungicide, immunomodulation, biological control

## Abstract

Although the vast majority of biological control agents is generally regarded as safe for humans and environment, the increased exposure of agriculture workers, and consumer population to fungal substances may affect the immune system. Those compounds may be associated with both intense stimulation, resulting in IgE-mediated allergy and immune downmodulation induced by molecules such as cyclosporin A and mycotoxins. This review discusses the potential effects of biocontrol fungal components on human immune responses, possibly associated to infectious, inflammatory diseases, and defective defenses.

## Introduction

Phytopathogenic microorganisms are related to infestation of several crops resulting in economic losses. The Peruvian fungal-like oomycete *Phytophtora infestans* a potato pathogen caused the Irish famine and diaspora in the XIX century (Abad and Abad, 1997; Axel et al., 2012; Yoshida et al., 2013). More recently, in Bahia—Brazil, the fungus *Moniliophtora perniciosa*, etiological agent of witch's broom, caused extensive economic losses in cocoa crops (Meinhardt et al., 2008; Teixeira et al., 2015). The *Trichoderma stromaticum* spores comprise effective biocontrol agent for *M. perniciosa* (de Souza et al., 2006). Although most microbiological control agents are generally recognized as safe to humans and environment (Wang et al., 2004; Mommaerts et al., 2009), some studies demonstrated that those agents imbalance mammalian immune system leading to diseases such as allergy (Halpin et al., 1994). Given the socioeconomic impact of monocultures (Roossinck and García-Arenal, 2015), infections and the environmental hazards of chemical pesticides, biocontrol agents emerge as a strategic option and their increased use may cause higher exposure of cocoa workers. Moreover, the high fungal persistence in the environment (Scheepmaker and Butt, 2010), mainly spores (Darbro and Thomas, 2009), potentially causes exposures of consumer population to fungal substances. Here, we review the eventual mammalian immunological interactions triggered by fungal components and the association with infective and inflammatory diseases.

## Agricultural biocontrol agent

Biopesticides comprise more selective activity against pests whereas chemical pesticides are associated to pest resistance induction, requiring more applications, and residual toxic effects (Berg, 2009). The multiple modes of action of most microbial biocontrol agents cause reduced resistance selection by pathogens, insects, or weeds (Gardener and Fravel, 2002; Alabouvette et al., 2006). The ability of biocontrol agents to grow and reproduce, surviving for prolonged periods in the environment generally in symbiotic consortia with hosts, contributes to applications of lower bioagent amounts (Whipps, 2001). The decreased residual effect of biocontrol agents compared to chemical pesticides is also related of effective agents employing small quantities diminishing environment exposure (Thakore, 2006; Gupta and Dikshit, 2010). Moreover, these biological agents, take part of the ecosystem, reducing the impact of their insertion in microbial community (Vázquez et al., 2000; Tahat et al., 2010).

The biocontrol agents are classified as microbial and biochemical pesticides (FAO, 1988). Microbial agents encompass viruses or live bacteria, fungi, and protists (Chandler et al., 2011). Biocontrol microbial agents may undergo different genetic modifications to optimize their biopesticide activity (Chet and Inbar, 1994; St Leger and Wang, 2010; Kowsari et al., 2014). On the other hand, biochemical agents are characterized by non-live parts of microbes such as single molecules or mixtures with pesticide activity, including enzymes, and other macromolecules (Chavan and Deshpande, 2013), hormones (Martínez-Medina et al., 2014), and metabolites (Degenkolb and Vilcinskas, 2016).

Around 135 products are commercialized worldwide as biocontrol agents (Chandler et al., 2011), including products from 13 fungi. Some fungal biopesticide species are summarized on Table 1. These species display variable pesticide action modes including parasitism of plant-infecting nematodes by *Paecilomyces* genus (Cabanillas and Barker, 1989; Castillo Lopez et al., 2014), colonization of insect's body cavities by several Hypocreales species from Ascomycota causing the host death (Tartar et al., 2005), environmental competition, parasitism with others undesirable fungi, and stimulation of defense mechanisms of plants by *Trichoderma* genus (Benítez et al., 2004). In addition, several fungal endophytic species colonize internal plant tissues, stimulating an important host defense mechanisms against pathogens (Wani et al., 2015). Some fungal species show a strong specificity for plant host, but analysis of host-specificity is complex and misleading because *in vitro* experiments do not completely simulate the natural environments (Stoeva et al., 2012). Furthermore, this organism group presents significant potential impact on the human and animal health due to propagation of spores adapted to dispersion for resisting harsh environmental conditions (Baxi et al., 2016).

**Table 1:** **Immunomodulatory biomolecules in biocontrol fungal**.

**Specie fungal biocontrol**	**Phytopathogen**	**Crop**	**Immunomodulatory biomolecules**	**References**
*Beauveria bassiana*	*Bemisia tabaci Hedypathes betulinus Tetranychus urticae*	*Chrysanthemum*, citrus, horticulture, cucumber; *Eucalyptus*, papaya, coffee, soybean		Tartar et al., 2005
*Cladosporium* sp.	*Uromyces appenciculatus Cronartium flaccidum Peridermium pini*	Beans, coffee, rice	β-glucan	Van Dyken et al., 2011
*Paecilomyces* sp.	*Citrus psyllid Spider mite, Thrips, Whitefly*	Apples and stonefruits, citrus, grapes, tree nuts, strawberries, melons, cucurbits, herbs, spices, beans		Cabanillas and Barker, 1989; Castillo Lopez et al., 2014
*Trichoderma polysporum*	*Fusarium, Phytopthara, Scelerotia*,	Vegetables, fruits and berries herbs and spices, ornamentals, Turf, forestry	Ciclosporin A	Dreyfuss et al., 1976; Benítez et al., 2004; Azam et al., 2012
*Trichoderma harzianum*				
*Trichoderma harzianum*	*Sclerotina, Fusarium, Rhizoctonia*	Bean, soy, corn, strawberry, vegetables, ornamentals	Gliovirin	Benítez et al., 2004; Rether et al., 2007
*Trichoderma virens*	*Sclerotium rolfsii, Rhizoctonia solani, Pythium* spp.	Sweet potato, pumpkin, corn, wheat, peanut, Soybean seed, cotton seedlings and Horticultural crops	Gliotoxin	Brian and Hemming, 1945; Lumsden et al., 1992; Benítez et al., 2004; Becker et al., 2016
*Trichoderma viride*				
*Trichoderma stromaticum*	*Moniliophthora perniciosa*	Cacao	Spores	de Souza et al., 2006; Alves-Filho et al., 2011

## Biocontrol agents and human health

Although fungal biocontrol pose numerous benefits, these agents can survive and reproduce in the environment, and so they are aspirated or swallowed by humans or other animals (Hansen et al., 2010; Luangsa-Ard et al., 2011). The increased exposure to fungi or fungal molecules may affect human health (Eduard et al., 2001). For instance, it is known that some fungi used as biocontrol agents can compromise the respiratory tracts of mammals (Madsen et al., 2007). Infections by biocontrol agents, considered opportunistic pathogens are common in immunocompromised patients, mainly submitted to immunosuppressive therapy such as organ transplant recipients. Disorders such as sinusitis and pulmonary lesions caused by *T*. *longibrachiatum* and *T*. *harzianum* were reported in intestine, liver and bone marrow transplant recipients especially in neutropenic patients (Furukawa et al., 1998; Guarro et al., 1999; Richter et al., 1999). *T. longibrachiatum* is the most common species involved in *Trichoderma* infections (Trabelsi et al., 2010) and its virulence factors include mycelial growth at 37 °C and physiological pH, hemolytic activity and toxicity to mammalian cells (Antal et al., 2005), extracellular protease(s) (Kredics et al., 2004) as well as resistance to antifungal compounds such as fluconazole, itraconazole, and amphotericin B (Singh et al., 1997; Richter et al., 1999; Espinel-Ingroff, 2001; Dóczi et al., 2004). Nevertheless, *T*. *longibrachiatum* was detected in sphenoidal sinus infection in immunocompetent patient, only displaying eosinophilia (Molnár-Gábor et al., 2013). Experimental models with rodents are used to measure potential effects such as allergenicity, toxicity, infectivity, and pathogenicity, in order to evaluate biofungicide safety (US Environmental Protection Agency (EPA), 1996).

Methods presented in the protocol of The Microbial Pesticide Test Guidelines of the Environmental Protection Agency of the USA (US Environmental Protection Agency (EPA), 1996) that evaluate risks to humans and domestic animals are carried out using live microorganisms such as *Bacillus thuringiensis* var *israelensis* SH-14 in rats, *Beauveria bassiana*, and *Paecilomyces fumosoroseus* in mice (Mier et al., 2005; Zimmermann, 2007; Mancebo et al., 2011). Despite the rigorous risk assessment protocol, epidemiologic studies have previously demonstrated the correlation of exposure to fungal organisms and frequency of diseases. For instance, the increased exposure to spores and mycotoxins from *Cladosporium* species affects alveolar type II cells, macrophages, and pulmonary surfactant production and composition (Kuhn and Ghannoum, 2003). Human cells exposure to *Cladosporium* extracts *in vitro* induces cytokines of Th1 and Th2-type Thelper cell and eosinophils migration (Shin et al., 2004). The exposure to 1500 *Cladosporium* spores/m^3^ reduced lung function in schoolchildren and the changes appear to be associated with the small size of the spores that are deposited in the human lower respiratory tract, and to *Cladosporium* allergens (Chen et al., 2014). Among 389 patients with suspected respiratory allergy and exposure to *T. harzianum* (Das and Gupta-Bhattacharya, 2009), 105 showed positive skin reaction against *T. harzianum* extract and IgE specific to fungal proteins.

## Biocontrol agents and immune system homeostasis

Exposure to high concentrations of environmental fungal spores can cause human disorders such as allergies and toxic mold syndrome (Edmondson et al., 2005; Eduard, 2009). In agriculture, the application of biocontrol products containing microbiological pest control agents (MPCAs) can increase the exposure of workers to microbial agents (Hansen et al., 2010). The exposure to indoor fungal spores and humidity seem to be associated with an increased risk of asthma morbidity in young children as well as people who have previously suffered asthmatic attack (Baxi et al., 2016). Nevertheless, the importance of exposure time, the potential of different fungal species and molecular components responsible for damage and symptoms are still unknown. A common mechanism associated with allergy triggered by biocontrol agents such as *B. bassiana* (Westwood et al., 2005, 2006), *Metarhizium anisopliae* (Ward et al., 2011), *Paecilomyces* and *Trichoderma viride* (Beezhold et al., 2008), and *Penicillium oxalicum* (Kochar et al., 2014) is the production of IgE against fungal molecules observed in animal models and human patients.

In contrast to immune response exacerbation due to stimulation of IgE production by common fungal allergens, a few studies demonstrated that biocontrol agents can impair immune system homeostasis through negative modulation. Mice exposed to intranasal *T. stromaticum* spores employed in *ex vivo* assays for cytokine measurements, revealed diminished IL-10 and IFN-γ levels in bronchoalveolar lavage fluid and splenocyte cultures (Alves-Filho et al., 2011). Besides that, phagocytes obtained from thioglycolate-treated mice, exposed to *T. stromaticum* spores *in vitro* showed downregulated production of nitric oxide (NO) by inducible nitric oxide synthase (iNOS) and reactive oxygen species (ROS) by neutrophils. In addition, both cell types display decreased expression of *Clec7a* gene that codes the Dectin-1 receptor, Toll Like Receptor 2 (*Tlr2*), and Toll Like Receptor 4 (*Tlr4*). Hence, the *in vitro* and *in vivo* experiments carried out with *T. stromaticum* suggested a possible negative modulation mainly of the cell components of the murine innate immune system.

Some molecules from biocontrol fungal agents, that act during the mycoparasitism or stimulating the defense mechanisms of the host plant, have been previously identified as modulators of the mammalian immune response (Table 1). For instance, *Cylindrocapon lucidum, Trichoderma polysporum* currently identified as *Tolypocladium inflatum, Fusarium oxysporum*, and *T. harzianun* fungi produce cyclosporin A (CsA) (Dreyfuss et al., 1976; Rodríguez et al., 2006; Azam et al., 2012). This molecule is considered a virulence factor for including its antifungal activity against the phytopathogen *M. perniciosa*, and one of its intracellular targets is cyclophilin protein. CsA activity assays over the pathogen *M. perniciosa* demonstrated inhibition of basidiospore germination and mycelium growth *in vitro* (Monzani et al., 2011). On the other hand, the immunomodulatory function of CsA, due to its well-established capacity to inhibit calcineurin, is associated to impaired activity of the nuclear factor of activated T cells (NFAT) and reduced activation, proliferation, and survival of lymphoid cells (Rovira et al., 2000). This immunosuppressant action of CsA is interesting because of its potential use for preventing acute rejection in organ transplantation (Borel et al., 1976; Levy, 2001). Another important action mechanism of CsA consists of inhibition of NO production by destabilization of the iNOS mRNA (Hämäläinen et al., 2009). Furthermore, CsA downregulate the signaling pathway of the NFκb transcription factor through the inhibition of TLR4 expression (Dusting et al., 1999; Rovira et al., 2000; Ge et al., 2012). The impact of CsA from environmental fungi in human health remains inconclusive since it is detected in crops such as maize (Mogensen et al., 2011), but the molecule displays slow and incomplete oral absorption (Ptachcinski et al., 1986). Detailed studies are required to clarify whether cyclosporin A produced by biocontrol fungi accumulate in cultures and if the consumption of these cultures comprises risks to the homeostasis of the human immune system.

**Figure 1:**
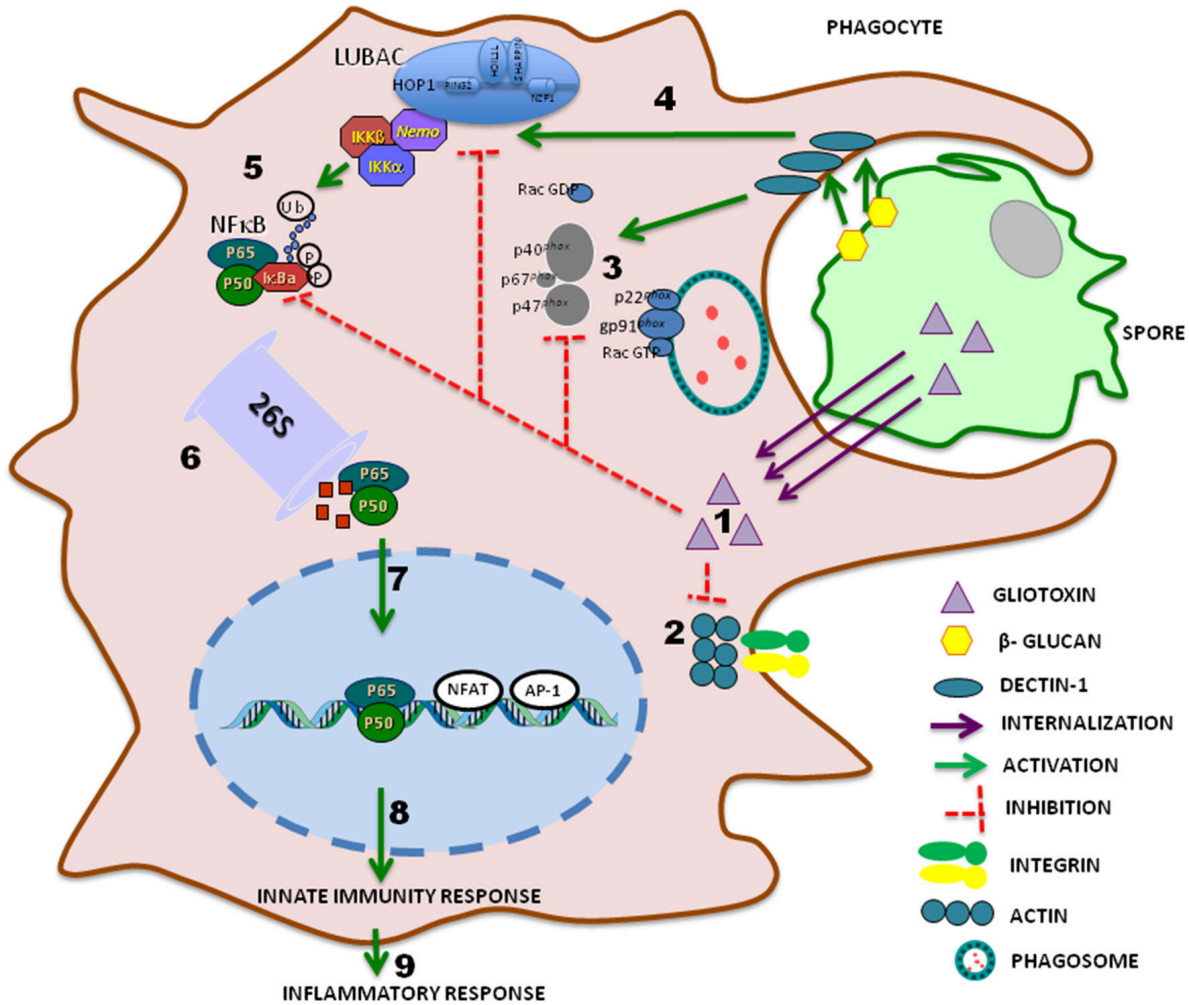
**Schematic representation of major phagocyte modulation pathway induced by gliotoxin from fungal: spore internalization by phagocytes is an essential mechanism to prevent hyphae formation by pathogenic fungi**. Gliotoxin triggers (1) F-actin disorganization, inhibition of phagocytosis by deregulation of PtdIns (3,4,5)P3 turnover, resulting in integrin and actin cytoskeleton dysfunction, preventing pseudopodia emission (2). Gliotoxin also blocks translocation of cytosolic phox proteins (p40, p67, p47) that bind to membrane proteins of phagolysosomes, gp91 and p22 to form NADPH oxidase, inhibiting reactive oxygen species (ROS) formation (3). Dectin-1-mediated ubiquitin chain formation (4) and NF-κB activity (5) are negatively modulated by gliotoxin. Phosphorylation of IκBα leads to its ubiquitination and proteasomal degradation (6). Active heterodimer p50-p65 is then released and translocated to the nucleus (7), binds to specific κB sites and either alone or in combination with other transcription factors, activates NF-κB target gene expression of the innate (8) and inflammatory immune response (9).

In addition to cyclosporin, peptaibiotics, siderophores, and epidithiodioxopiperazine (ETPs) make up the group of secondary metabolites named non-ribosomal peptides (NRPs) and, the success of *Trichoderma* species as biocontrol agent is at least in part due to the ability of these fungi to produce these biotechnology relevant metabolites (Zeilinger et al., 2016). Particularly the toxicity of the ETPs gliovirin and gliotoxin is due to the eventual inactivation of proteins by interaction of their disulfide bridges to thiol groups (Gardiner et al., 2005). In mammals, modulation of the immune response by gliovirin is related to decreased expression of tumor necrosis factor in consequence of inhibition of the AP1 and NFκb-factors transcription (Rether et al., 2007). In addition, gliotoxin, a well-studied metabolite, inhibits several mechanisms of innate immunity including phagocytosis, activation of the NADPH oxidase complex responsible for the generation of ROS and NFκB nuclear factor, indispensable for the production of cytokines and reactive nitrogen species such as nitric oxide (Figure 1; Lumsden et al., 1992).

Gliotoxin was firstly identified in *Trichoderma virens* (Brian and Hemming, 1945; Lumsden et al., 1992) and is produced by several fungal species (Scharf et al., 2016). This molecule showed antibiotic activity against plant pathogens such as *Rhizoctonia solani, Pythium ultimum, Sclerotinia sclerotiorum* (Vargas et al., 2014), and *Botrytes cinerea* (Lorito et al., 1994). The action mechanisms against phytopathogens include cytoplasmic material leakage (necrosis), inhibition of sporangia germination, and mycelial growth (Roberts and Lumsdem, 1990; Lorito et al., 1994; Lewis et al., 2005; Scharf et al., 2016).

Although there is little evidence of the involvement of gliotoxin in human disease, exposure to this toxin appears to occur during infections by pathogenic fungi such as *Aspergillus fumigattus* and *Candida albicans*, but evidence of immunosuppressive activity associated with intoxication due to fungal infection remains inconclusive (Bondy and Pestka, 2000). The antigen-presenting cells including thioglycolate-elicited mouse peritoneal macrophages exposed to gliotoxin show inhibited phagocytosis and adhesion capacity (Müllbacher and Eichner, 1984). J774 cells and human macrophages differentiated from THP1 monocytes showed increased phagocytosis of *A. fumigatus gliP*Δ mutant conidia, a strain depleted of the *gliP* gene, responsible for the biosynthesis of gliotoxin, as compared to wild-type *A. fumigatus* conidia and conidia gliPR (reconstituted gliP). Both J774 and THP1 incubated with exogenous gliotoxin, display significantly reduced uptake of conidia of the three strains, indicating that the gliotoxin produced by *A. fumigatus* inhibits phagocytosis by macrophages (Jia et al., 2014). Low concentrations of gliotoxin (30–100 ng/mL) inhibit zymosan phagocytosis by human polymorphonuclear leukocytes. In addition, the compound induces neutrophil cell shrinkage, F-actin collapse in the perinuclear region and disappearance of filopodia without affecting the protein polymerization process, but this reorganization does not seem to correlate with phagocytosis reduction (Coméra et al., 2007). Recently it has been shown that gliotoxin affects phagocytosis, a key macrophage function, modifying the homeostasis of phosphatidylinositol 3,4,5-trisphosphate and interfering in integrin activation and actin dynamics (Schlam et al., 2016; Figure 1).

This gliotoxin inhibits neutrophil ability to produce ${\text{O}}_{2}^{•-}$, especially when added prior to the activation of the NADPH oxidase by phorbol myristate acetate (PMA; Yoshida et al., 2000; Tsunawaki et al., 2004). This inhibition is a consequence of the reduction of translocation levels of cytosolic Phox components to membrane rather than of oxidase assembly (Tsunawaki et al., 2004; Figure 1). The immunoregulatory effects of gliotoxin on mononuclear cells are due at least in part, to their potential to block the degradation of the most abundant inhibitory subunit of nuclear factor κB (NF-κB), IκBα (Figure 1; Pahl et al., 1996; Kroll et al., 1999). Recently the high throughput screening (HTS) technique with Tb3+ -Fluorescein FRET was used to demonstrate that gliotoxin selectively binds at the catalytic center of the linear ubiquitin chain assembly complex (LUBAC) inhibiting ubiquitin chain formation and signal-induced NFκB activation (Sakamoto et al., 2015). The reduction in IκBα degradation results in inhibition of cytoplasmic activation and nuclear translocation of NFκB in different cells. Gliotoxin induces negative modulation of pro-inflammatory cytokines associated with down-regulation of genes, which in turn are associated with inhibition of NFκB, and result in increased susceptibility to microorganisms (Kupfahl et al., 2006). In human monocyte cell lines, gliotoxin favors a cytokine imbalance with relevant inhibition of IL-10 production (Johannessen et al., 2005).

The detection and relevance of gliotoxin from biocontrol fungi to human health, particularly for occupationally exposed individuals were scarcely studied. There is no in-depth, *bona fide* knowledge regarding symptoms and doses that cause susceptibility in individuals and little is known about the presence of this toxin in bioaerosols. Studies determined a total of 0.22 microgram of gliotoxin in extract of 6.2 × 10^8^ spores from *Aspergillus fumigatus* mechanically disintegrated (Schulz et al., 2004). The presence of gliotoxin has been described in biocontrol fungal species of the genus *Trichoderma* including *T. viride* and *T. virens* (Brian and Hemming, 1945; Anitha and Murugesan, 2005). Thus, inhalation of aerosols containing high concentrations of spores may comprise a potential health hazard.

Cell wall components such as chitin and β-glucan were identified in biocontrol fungi spores and are extensively studied during biocontrol-pathogen-plant interaction. These carbohydrate polymers may induce modulatory activity affecting the production of both pro-inflammatory and anti-inflammatory cytokines (Sorrell and Chen, 2009; Koller et al., 2011; Brodaczewska et al., 2015; Becker et al., 2016). Chitin a polymer of beta-(1,4)-linked N-acetylglucosamine (GlcNAc) and β-glucan, glucose polymers linked together by1-3 linear β-glycosidic are pathogen-associated molecular patterns (PAMPs) able to modulate the innate immune response of various cells including monocytes, macrophages, neutrophils, and NK through of pattern recognition receptors (PRRs), including TLR-2 and C-Type Lectin Receptors (CLR) such as Dectin-1, Dectin-2, and Manose Receptor (Barreto-Bergter and Figueiredo, 2014; Brodaczewska et al., 2015). These interactions between PAMPs and specific PRRs upregulate innate responses and Th1 responses in humans and animals (Rop et al., 2009; Muzzarelli, 2010). However, chitin is able to induce the Th2 immune response, exacerbating allergic reactions (Gregory and Lloyd, 2011; Dubey et al., 2015). In addition, soluble β-glucan from *Candida albicans* reverses or impairs the activation of human monocytes cultured with endotoxin. This β-glucan effect is associated to the suppressed production of the type 1 cytokines IL-2 and IFN-γ by cultured human PBMC (Nakagawa et al., 2003). The dual immunological effect de chitin and β-glucan is due at least in part to features such as particle size, tissue in which the contact with macrophage takes place, environmental cytokines and surface availability of spore β-glucans (Da Silva et al., 2009; Mintz-Cole et al., 2013; Alvarez, 2014). Furthermore, chitin induces accumulation of innate immune cells expressing IL-4 including eosinophils and basophils in tissue from mice and these events are associated with allergy (Reese et al., 2007). Specifically, fungal chitin from dust collected from the homes of asthmatic individuals, induces marked eosinophilic lung infiltration particularly whenever associated with β-glucans (Van Dyken et al., 2011).

As the structure of the fungal cell wall and the PAMPs exposed at the cell surface are genus-, species- and morphotype-dependent (Brodaczewska et al., 2015), detailed studies on modulation of the mammals immune system induced by biocontrol agent spores and mycotoxins is relevant to medicine and biotechnology. The compromised immune system may favor the development of opportunistic pathogens (Fishman, 2011) and even neoplastic diseases (Barle et al., 2014). The immunosuppressive molecules may comprise chemotherapy agents for autoimmunity and hypersensitivity reactions (Thell et al., 2014). Although there is little research approaching the health effects of biocontrol fungi, the preliminary data indicate that the impact upon immune system can be more significant than previously supposed. Henceforth, further studies are required to identify the compounds of these fungi, accumulation during crop storage, amount ingested or inhaled by the consumer/worker, mechanisms associated with immune modulation, eventual health hazards as well as potential biotechnological use of such compounds.

## Author contributions

All authors listed, have made substantial, direct and intellectual contribution to the work, and approved it for publication.

## Funding

This article was funded by a grant from Universidade Estadual de Santa Cruz—Brazil.

### Conflict of interest statement

The authors declare that the research was conducted in the absence of any commercial or financial relationships that could be construed as a potential conflict of interest.
